# Identification and characterisation of midbrain nuclei using optimised functional magnetic resonance imaging

**DOI:** 10.1016/j.neuroimage.2011.08.016

**Published:** 2012-01-16

**Authors:** Eve H. Limbrick-Oldfield, Jonathan C.W. Brooks, Richard J.S. Wise, Francesco Padormo, Jo V. Hajnal, Christian F. Beckmann, Mark A. Ungless

**Affiliations:** a Medical Research Council Clinical Sciences Centre, Imperial College London, Hammersmith Hospital, Du Cane Road, London, W12 0NN, UK; b Centre for Functional Magnetic Resonance Imaging of the Brain (FMRIB), Nuffield Department of Clinical Neurosciences, University of Oxford, John Radcliffe Hospital, Oxford, UK; c Division of Experimental Medicine, Imperial College London, Hammersmith Hospital, Du Cane Road, London W12 0NN, UK; d Donders Institute, Radboud University Nijmegen and MIRA Institute, University of Twente, The Netherlands

**Keywords:** PNM, physiological noise model, RETROICOR, retrospective image correction, fMRI, Midbrain, Superior colliculi, Physiological noise, Registration

## Abstract

Localising activity in the human midbrain with conventional functional MRI (fMRI) is challenging because the midbrain nuclei are small and located in an area that is prone to physiological artefacts. Here we present a replicable and automated method to improve the detection and localisation of midbrain fMRI signals. We designed a visual fMRI task that was predicted would activate the superior colliculi (SC) bilaterally. A limited number of coronal slices were scanned, orientated along the long axis of the brainstem, whilst simultaneously recording cardiac and respiratory traces. A novel anatomical registration pathway was used to optimise the localisation of the small midbrain nuclei in stereotactic space. Two additional structural scans were used to improve registration between functional and structural T1-weighted images: an echo-planar image (EPI) that matched the functional data but had whole-brain coverage, and a whole-brain T2-weighted image. This pathway was compared to conventional registration pathways, and was shown to significantly improve midbrain registration. To reduce the physiological artefacts in the functional data, we estimated and removed structured noise using a modified version of a previously described physiological noise model (PNM). Whereas a conventional analysis revealed only unilateral SC activity, the PNM analysis revealed the predicted bilateral activity. We demonstrate that these methods improve the measurement of a biologically plausible fMRI signal. Moreover they could be used to investigate the function of other midbrain nuclei.

## Highlights

► Functional MRI of the midbrain is difficult because it is small and prone to noise. ► Our midbrain optimised group registration allows accurate localisation of activity. ► We model and remove the structured physiological noise in the data. ► These optimisations improve the detection of a visually induced midbrain signal. ► These methods are automated and are applicable to any midbrain nuclei.

## Introduction

There is increasing interest in extending functional magnetic resonance imaging (fMRI) to the study of human brainstem neural activity, particularly in the midbrain due to its role in modulating behaviour. Animal studies indicate that the midbrain is involved in many functions, including visual perception (Cynader and Berman, 1972; Goldberg and Wurtz, 1972; Schiller and Koerner, 1971), reward processing (Schultz et al., 1997), and nociception (Basbaum and Fields, 1984). However, relatively little is known about how these results may translate to humans. This is due to the fact that midbrain signals are difficult to detect and quantify using non-invasive in-vivo neuro-imaging techniques. This is due to the difficulty in obtaining a reliable blood oxygen level dependent (BOLD) signal, the indirect measure of neural activity utilised by fMRI, from the human midbrain.

It is challenging to measure a BOLD signal from this area for two main reasons. Firstly, the nuclei within the midbrain are small and tightly packed. In order to localise signals at a group level within one of these structures, accurate registration to a standard brain template must be carried out. However conventional registration methods used for fMRI are optimised for whole-brain data sets, and do not optimise for maximum accuracy of midbrain registration. Secondly, the midbrain is prone to artefacts from the cardiac (Dagli et al., 1999; Greitz et al., 1992; Poncelet et al., 1992) and respiratory (Raj et al., 2001) cycles, which add structured noise to the data. We designed this study to address these challenges, using novel registration methods combined with physiological noise modelling to optimise for signal identification in midbrain fMRI.

### Registration

In cognitive neuroimaging the process of ‘registration’ typically involves transforming functional data to a standard space template to facilitate between-subject group analysis. In conventional whole-brain fMRI this is usually achieved in two steps: the functional data, which has limited structural information, is first transformed onto a high-resolution structural image, which is in turn transformed onto a standard brain template. These two steps are then concatenated and applied to the fMRI data.

Midbrain fMRI studies have used this conventional approach using either a T1-weighted (T1w) structural image (Krebs et al., 2010; Sigalovsky and Melcher, 2006; Zhang et al., 2006) or a proton density structural image (Dunckley et al., 2005). However there are two reasons to suggest that such methodology does not lead to robust midbrain registration. Firstly, fMRI optimised for the midbrain typically has a limited field-of-view (FOV). This is because high-resolution functional scans are required to accurately localise activity to a specific midbrain nucleus, so a long repetition time (TR) would be required to collect data from the whole brain. In order to fit an experiment within a reasonable scan time and maintain temporal resolution, data are collected from slices over the midbrain only. Within this limited FOV there is less structural information than would be available in a whole-brain FOV, so the transformation of the functional data onto the high-resolution structural image is not as reliable or as accurate as the whole-brain equivalent. Secondly, registration accuracy in midbrain fMRI needs to exceed the accuracy that would normally be expected with whole-brain fMRI. This is due to the smaller size of the midbrain nuclei, and the close proximity of the nuclei to each other.

In recognition of the challenges facing midbrain registration, many fMRI studies have circumvented the need for registration completely and used a region-of-interest (ROI) approach. Voxels within individually defined ROIs are averaged and these averages are compared at a group level (DuBois and Cohen, 2000; Guimaraes et al., 1998; Hawley et al., 2005; Schneider and Kastner, 2005; Topolovec et al., 2004; Tracey et al., 2002; Wall et al., 2009). However, ROI analyses rely strongly on a-priori predictions, so may miss unexpected results. Furthermore, the inclusion of non-active voxels in the ROI average can remove genuine effects, and increase the likelihood of accepting false negatives. Alternatively, manual registration has been used for midbrain fMRI (Sylvester et al., 2007) but this is a time consuming method and may be vulnerable to investigator bias. Automated linear registration of structural images into standard space has been optimised for the midbrain (Napadow et al., 2006; Pattinson et al., 2009). However the use of non-linear registration methods may circumvent the need for this optimisation, as these methods apply transformation to standard space at a local level, rather than applying the same transformation to the whole brain, as is the case with linear registration (Klein et al., 2009).

Here we present an unbiased, user independent and novel registration pathway that improves on conventional registration to increase the accuracy with which functional data are transformed onto the standard brain template at the level of the midbrain. This is achieved with the addition of two intermediate whole-brain structural scans prior to transformation of the data to a T1w high-resolution structural image. Functional data are first transformed onto a whole-brain echo-planar image (EPI) that matches the functional data, but with full field-of-view. This overcomes the problem created by having only a limited number of slices to drive registration. The resulting data is then transformed onto a high-resolution T2-weighted (T2w) image. Both the functional images and T2w images contain areas of low image intensity that correspond to the red nuclei (RN) and substantia nigra (SN), due to their high iron content (Drayer et al., 1986). The intensity boundaries surrounding these areas in the midbrain can then be utilised to drive registration algorithms. If a T1w image were used as the initial high-resolution structural image, only the edges of the midbrain could be used for registration as there is uniform signal throughout the midbrain structures with such a T1w sequence. Thus, accuracy within the midbrain would be compromised. Further, we propose to weight the cost function evaluation within the registration algorithm towards accurate sub-cortex registration (at the expense of accuracy with respect to registration of cortex). The evaluation study was designed to demonstrate the accuracy of this method at the level of the midbrain, compared with the accuracy of conventional registration methods.

### Physiological noise

One additional major challenge facing midbrain fMRI is that, due to its anatomical location, it is prone to physiological artefacts. During the cardiac cycle the midbrain undergoes a bulk motion in the direction of the foramen magnum, due to the increased intracranial pressure as blood enters the brain (Poncelet et al., 1992). Such bulk motion causes spatio-temporal blurring of the BOLD signal across voxels. Also the large blood vessels adjacent to the midbrain are subject to cardiac pulsality (Dagli et al., 1999; Greitz et al., 1992) causing BOLD signal intensity changes in nearby tissue. Furthermore, intracranial pressure changes and pulsatile movement of blood vessels produce oscillatory motion in the cerebrospinal fluid (CSF) surrounding the brain and brainstem (Friese et al., 2004; Klose et al., 2000), which gives rise to in-flow signal artefact on the EPI typically used to record functional information (Piché et al., 2009). In addition to cardiac related artefacts, the respiratory cycle also causes bulk magnetic susceptibility changes within the brain tissue during the respiratory cycle (Raj et al., 2001). There is also a significant interaction between these two sources of noise (Brooks et al., 2008; Harvey et al., 2008).

In order to reduce the interference of physiological noise, many midbrain fMRI studies use cardiac gating (D'Ardenne et al., 2008; DuBois and Cohen, 2000; Guimaraes et al., 1998; Hawley et al., 2005; Napadow et al., 2009; Sigalovsky and Melcher, 2006; Zhang et al., 2006). In this approach, imaging data is collected in between heartbeats, assuming that the brain is relatively stable during this time. This limits the number of slices that can be collected per volume. In addition, the TR that results from the variable heart rate causes differences in the T1 relaxation (Guimaraes et al., 1998) that requires correction. Most importantly, this approach does not correct for respiratory artefacts or the noise resulting from an interaction between the cardiac and respiratory cycles.

Alternative methods have been developed that use physiological measures to model and remove structured noise from fMRI data (Glover et al., 2000; Hu et al., 1995; Liston et al., 2006). Retrospective Image Correction (RETROICOR) (Glover et al., 2000) was originally developed for whole-brain fMRI, and physiological noise is removed by first assigning a cardiac and respiratory phase to each slice of data based on its acquisition time relative to the physiological cycles, then modelling their likely effect on imaging data using a basis set including four Fourier terms. Here we use a modified version of RETRICOR, the Physiological Noise Model (PNM), which is implemented via the general linear model and therefore avoids problems relating to adjusting variance estimates for the loss of degrees of freedom when pre-filtering. The PNM was developed for spinal (Brooks et al., 2008) and brainstem (Harvey et al., 2008) studies. In the brainstem a significant amount of noise is generated by an interaction between the cardiac and respiratory cycles, which can be successfully modelled with the PNM (Harvey et al., 2008; Pattinson et al., 2009). In addition, low frequency fluctuations in the heart rate may produce low-frequency noise in fMRI data (Chang et al., 2009), and this is also accounted for within the PNM.

In this study we applied the PNM to an fMRI experiment using visual stimulation, and compared the resulting activation with those from a conventional analysis. The visual stimulus was a moving black and white checkerboard, known to activate the superior colliculus (SC) in fMRI using an ROI approach (DuBois and Cohen, 2000; Schneider and Kastner, 2005) or manual registration (Sylvester et al., 2007). Therefore, any failure to detect a task-related BOLD signal change in the midbrain with this stimulus could primarily be attributed to sub-optimal processing.

## Methods

### MRI acquisition

Sixteen healthy subjects (seven female) aged between 22 and 60 years, with normal or corrected to normal vision, participated in this study. Two subjects were excluded due to poor shimming during acquisition of the functional data. Two subjects were excluded due to an inability to detect their cardiac signal above background noise. Two subjects were excluded because we failed to secure revisits for structural scans. The ten participants included in the analysis showed minimal movement during the functional scans (less than 1 mm), which improved the chances of good signal-to-noise (SNR) within the midbrain. MR scanning was performed on a 3T Philips Intera scanner with an eight-channel phased array head coil. A full table of acquisition parameters is available in the Supplementary materials. Subjects lay supine on the scanner, with padding underneath and surrounding the head. Physiological data were recorded using the scanner's in-built system. This included a vector-cardiogram (VCG) trace via electrocardiogram (ECG) pads on the chest, and a respiratory trace via a pneumatic belt. In order to synchronise the physiological data with the functional scans, the scanner's physiological recording software was modified to simultaneously record a trigger at the beginning of each slice acquisition.

Functional MR images were obtained using a T2*-weighted, EPI sequence with a FOV that covered the long axis of the brainstem (TE = 44 ms, TR = 1600 ms, flip angle = 90°; resolution, 1.5 × 1.5 mm; matrix size, 144 × 144; slice thickness, 1.5 mm; 16 coronal slices; no slice gap; interleaved slice order). A SENSE factor of two in the left-right direction was used to reduce susceptibility related artefacts in the data. Slices were aligned parallel to the anterior wall of the fourth ventricle. The slice orientation and placement was selected to minimise inhomogeneity in the main magnetic field (Dunckley et al., 2005). Immediately following the functional run, a whole-brain EPI scan was collected, using the same shim settings and voxel size as the functional run, but with more slices (147 slices; TE = 44 ms ;TR = 14.3 s). Two further functional data sets were collected, but are not reported here. A T2w structural scan (TE = 80 ms; TR = 2000 ms; resolution, 1.8 × 1.8 mm; slice thickness, 2.19 mm; 80 slices) and an MPRAGE T1w structural scan (resolution, 1.15 × 1.15 mm; slice thickness, 1.2 mm; 150 slices) were also obtained.

#### fMRI paradigm

During the functional scan a visual stimulus was repeatedly presented on a screen visible to subjects lying supine in the scanner. The stimulus was a smoothly rotating semi circle made of alternating black and white checks that scaled linearly with eccentricity. The checks reversed contrast at 8 Hz and the semi-circle rotated at 1 Hz. Each presentation lasted for 2 s, with a variable inter stimulus interval of between 1400 ms and 11000 ms. The trials were jittered. Stimuli were presented using the Psychophysics Toolbox extension (Brainard, 1997; Pelli, 1997) for MATLAB (2008b, Natick, Massachusetts; The Mathworks Inc.).

### Analysis

Data were analysed using the FMRIB Software Library (FSL) (http://www.fmrib.ox.ac.uk/fsl). Pre-processing of the functional data included motion correction to the mean volume using McFLIRT (Motion Correction FMRIB's Linear Image Registration Tool) (Jenkinson et al., 2002; Jenkinson and Smith, 2001), spatial smoothing (full width at half maximum = 2 mm), and high pass temporal filtering. BET (Brain Extraction Tool) (Smith, 2002) was applied to all brain images. Prior to model estimation using FEAT (FSL Expert Analysis Tool) v5.98, cardiac peaks (the R-wave) were extracted from the ECG trace, and high frequency scanner noise was removed from the respiratory trace. The regressors of the PNM were estimated from physiological data using a custom MATLAB routine (Brooks et al., 2008). Access to these files and instructions on implementing the PNM in FEAT are available (http://www.fmrib.ox.ac.uk/Members/jon/physiological-noise-correction).

#### Registration

All registration steps from functional space into T1w structural space were carried out using rigid body transformations in FLIRT (FMRIB's Linear Image Registration Tool) (Jenkinson et al., 2002; Jenkinson and Smith, 2001) whilst registration from T1w structural to standard space was carried out using FNIRT (FMRIB's Non-linear Image Registration Tool).

**Fig. 1:**
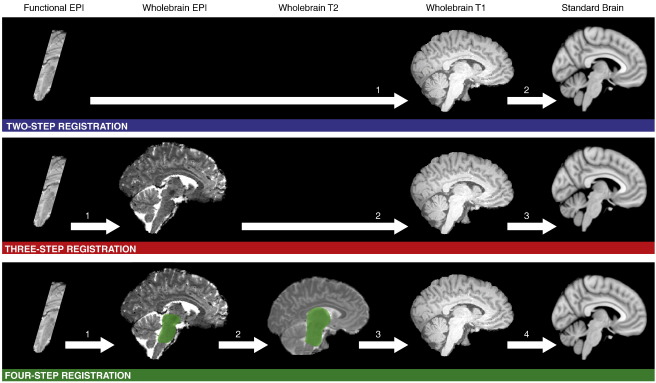
Three alternative registration pathways. Functional data was co-registered into MNI standard space using three alternative registration pathways. The presence of the green shaded areas in the four-step registration pathway indicates that a mask was used to weight the transform to the shaded area.

Three alternative registration pathways were tested for each participant (see Fig. 1). For the two-step registration pathway the mean functional image was registered to the T1w structural, this was then registered to the Montreal Neurological Institute (MNI) standard brain template. For the three-step registration pathway the mean functional image was first registered to the whole-brain EPI that matched the functional data in terms of contrast and resolution. This pathway is recommended by FSL (http://www.fmrib.ox.ac.uk/fsl/flirt/ztrans.html) as a way to improve the registration of data with a limited FOV. The whole-brain EPI was then registered to the T1w image and the T1w registered to the MNI template. Four-step registration started with the same transform of functional data to the whole-brain EPI. This step was then optimised for the midbrain: a hand drawn mask that covered the midbrain and pons of the EPI image was used to weight the transform for accuracy within these masked areas. Optimising a registration step in this way has previously been reported (Napadow et al., 2006; Pattinson et al., 2009). This corrected for any differences between the mean functional image and the whole brain EPI image due to head movement between the two scans. The second step was transforming the whole-brain EPI onto a T2w structural scan. As with the first step of this pathway, the transform was optimised using a weighting mask. The mask was in the T2w space and covered the thalamus, midbrain and pons. It was drawn once in standard space and transformed onto the individual T2w structural images. EPI images contain distortion in the phase-encode direction, so this adjustment to the transform allowed optimisation of the midbrain by ignoring areas of the brain subject to distortion. The third step was transforming the T2w image onto the T1w structural, a transform that needed no optimisation. The fourth step was transforming the T1w structural into the MNI template. In all cases, the initial steps (up to the T1w structural) were concatenated into a single transform before being applied to the functional data to avoid image degradation through multiple transforms.

**Fig. 2:**
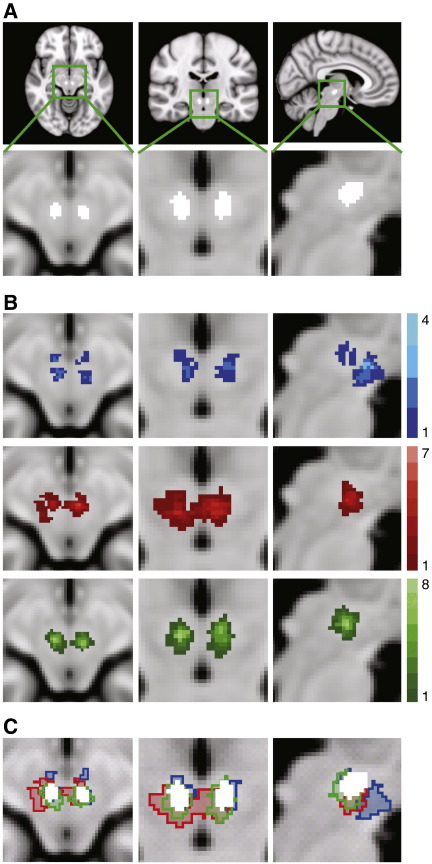
Assessing the accuracy of the registration pathways. All images are overlaid on an MNI T1 standard brain. (A) The standard location of the RN, derived from an MNI T2 template. The nuclei are shown in white in transverse, coronal and sagittal planes. (B) For each participant the RN were defined in functional space and then transformed into standard space using the three registration pathways. The group RN maps show the summation of all participants' RN in standard space for each registration pathway (blue = two-step registration, red = three-step registration, green = four-step registration). The legends indicate how many participants' nuclei overlap at each voxel. (C) The standard location of the RN is overlaid on the group RN maps as defined by the three registration methods. All images are shown in radiological convention.

To test the three registration pathways the RN was defined in each participant and transformed into standard MNI space using the transforms derived from the three registration pathways. This structure was selected as it was fully within the FOV of the functional scans, and was clearly identifiable using an automated method free from experimenter bias. No other areas of high contrast were suitable as they were not completely covered by the FOV (e.g. the SN), or were not definable using the automated method in a way that would ensure exactly the same structures has been selected for each participant. (e.g. the tissue-CSF boundaries). Also functional activity was not investigated within the RN in the fMRI task, so the assessment of the registration pathways was independent of the activation results. The RN was defined in the mean functional images using an automated tool that filled an area with a 3D mask, until a signal intensity change was detected (MRIcro 1.4, Chris Rorden, Georgia Institute of Technology, Atlanta, Georgia, http://www.cabiatl.com/mricro/). The standard location of the RN was identified using the same tool on a standard MNI T2w template (see Fig. 2A). Thus the location of the participant's RN in standard space, using three registration pathways, could be compared to the ‘gold standard’ of locating the RN on the T2w standard template.

#### fMRI analysis

Two sets of statistical analyses were carried out on the functional data, one with the PNM to remove physiological noise from the data, and one without. The analyses were identical, with the exception of the design matrix used in the general linear model (GLM).

To estimate and remove the influence of physiological noise from the time series data in the PNM analysis, we applied a modified version of RETROICOR (Glover et al. 2000), which models the cardiac and respiratory cycles using sine, cosine and interaction terms (Brooks et al., 2008). For each slice in the volume, a phase was assigned independently according to its acquisition relative to the cardiac and respiratory cycles. In total eight cardiac terms, eight respiratory terms, and sixteen interactions terms were used to model the structured physiological noise in the data. A heart rate regressor was also included (Chang et al., 2009). To remove the modelled noise from the data, these variables were included in the GLM. Removing structured noise from the data set in this way makes the detection of genuine effects more likely, and reduces the likelihood of accepting false positives (Harvey et al., 2008).

**Fig. 3:**
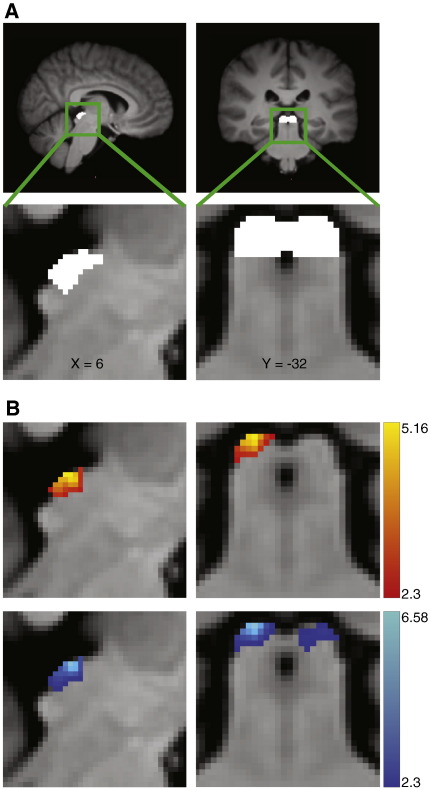
fMRI results. (A) The location of the SC, defined using anatomical boundaries on the group T1 standard brain, are shown in coronal and sagittal planes. (B) The SC responded to visual stimulation. Statistical maps computed from data without PNM revealed activity in the *right SC only*. The pattern of activation was modified when including a PNM, to include activity in the *SC bilaterally*. The statistical maps show significant clusters of voxels within a colliculi mask (determined using non-parametric permutation testing with a corrected threshold of p < 0.05 and a nominal T-value of 2.3) The T-values of the voxels within significant clusters are indicated by the legends. The top panel (red-yellow) shows unilateral SC activity revealed using a traditional analysis. The lower panel (blue-light blue) shows bilateral SC activity revealed using the PNM analysis.

For both analyses the first level of statistical analysis of the functional data (at the individual subject level) was carried out using a GLM approach. A model of the BOLD response to visual stimulation was constructed by convolving the stimulus input function with a gamma hemodynamic response function (HRF) with time-to-peak of 4 s. This short HRF has been shown to better represent the blood flow properties of the superior colliculi (Wall et al., 2009). A temporal derivative of the visual stimulation was also included, to allow for variation in individual HRFs. A single regressor that described global head motion was also included. For the PNM analysis, the 33 physiological regressors were also included in the GLM. Group statistics were carried out using FLAME (FMRIB's Local Analysis of Mixed Effects), (Beckmann et al., 2003; Woolrich et al., 2004). Variance that was explained by and unique to the visual regressor was represented as a statistical map, which was subsequently tested using non-parametric permutation testing to correct for multiple comparisons (Nichols and Holmes, 2002). This testing was carried out with RANDOMISE, part of FSL. All statistic images were cluster corrected (Worsley et al., 1992) to a significance level of p < 0.05 with a nominal T-value of 2.3 using standard cluster correction within RANDOMISE. Task activation was tested against an implicit ‘rest’ baseline. Prior to thresholding, a hand drawn mask was applied to the functional data to include only voxels from the superior and inferior colliculi. The inferior colliculi voxels were included to ensure that the visual response was correctly localised to the superior colliculi. The colliculi were defined on the MNI template using an anatomical atlas (Naidich et al., 2009). Fig. 3A shows the location of the SC on the MNI template.

## Results

### Registration

For each of the registration pathways a group RN mask was created by adding together all ten individual RN masks that had been transformed into standard MNI space. Fig. 2B shows the group RN mask for each of the registration pathways. Upon visual inspection, it is clear that the RN is poorly co-localised when the two-step registration pathway is used, with a maximal overlap of four individual RN masks. There is a marked improvement with the three-step registration with a greater maximal overlap of seven. However the four-step registration shows the greatest maximal overlap of eight, with a more symmetrical and tightly packed distribution of voxels. The maximal overlap was eight not because the procedure failed for two participants, but because there was individual variability in the location of the individual RN masks within the standard RN template. Using this registration procedure, each individual RN mask did overlap with the standard RN template. Comparing the location of the group RN masks (Fig. 2C) shows that the four-step registration results in the best co-localisation with the standard RN mask.

The registration pathways were assessed statistically using a repeated measure ANOVA. The number of voxels of the standard RN mask that were covered by the individual RN masks was significantly affected by the registration pathway used (F(2, 18) = 6.52, p < 0.05). Planned contrasts showed that the four-step registration pathway led to more overlap between the group RN mask and the standard RN mask than the two-step registration (F(1, 9) = 8.89, p < 0.05) and the three-step registration (F(1, 9) = 6.86, p < 0.05). Accuracy of the different registration pathways was also made on the basis of the number of voxels from the individual masks that fell outside the standard RN mask after normalisation to the template. There was a significant main effect of chosen registration pathway (F(2,18) = 6.33, p < 0.05). Planned contrasts showed the four-step registration pathway led to fewer voxels outside the standard RN mask than the two-step registration (F(1, 9) = 8.89, p < 0.05) and the three-step registration (F(1, 9) = 6.86, p < 0.05). Therefore, the fMRI data were analysed using the four-step registration method.

### fMRI

All statistic images were cluster corrected to a significance level of p < 0.05 with a nominal T-value of 2.3 using non-parametric permutation testing (RANDOMISE). The resulting statistical maps are shown in Fig. 3B. Conventional analysis revealed a significant response in voxels within the right SC alone. When the PNM was included in the GLM, visual activity was localised to the SC bilaterally. To check that possible left SC activity in the conventional analysis was not hidden due to conservative cluster thresholding, the analysis was repeated with a less conservative threshold (T > 1.83). Even with this low threshold (corresponding to uncorrected p < 0.05), no activity was revealed in the left SC without the PNM.

**Fig. 4:**
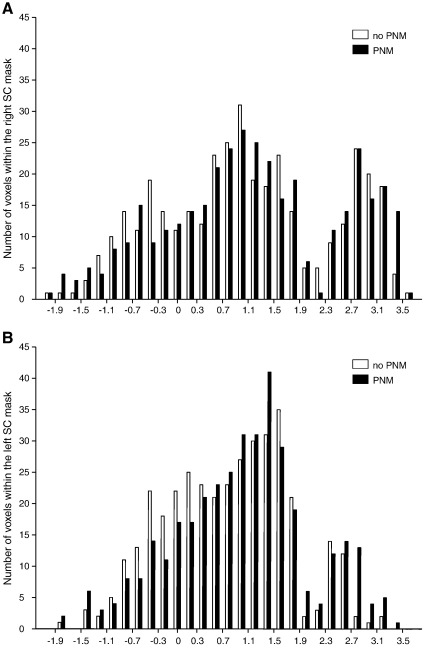
The distribution of Z-statistics in the SC revealed with the traditional and the PNM analysis. Histogram showing the Z-score distribution of voxels within the left and right SC for both analyses (white = conventional analysis, black = PNM analysis).

The distribution of the Z-scores of all voxels within the right and left SC in both the conventional and PNM analysis is shown in Fig. 4. There is an increase in the number of significant voxels in the right SC when the PNM is included in the GLM (from 103 voxels to 148). However the main effect of the PNM is the recovery of significant voxels in the left SC (83 voxels with PNM). However, this cluster extends by 28 voxels into the left inferior colliculus, so is not as well localised as the right SC activity.

To check that the PNM did not lead to further false positives outside of the colliculi, and to investigate the signal blurring that had occurred in the left inferior colliculus for the PNM analysis, we repeated the analysis using a mask that covered the entire midbrain. Using this larger mask we found clusters of activity adjacent to the left SC with both the conventional and the PNM analysis. In the case of the conventional analysis this cluster was adjacent to the left SC and extended across 56 voxels. For the PNM analysis this cluster extended from the left SC, but also included 46 non-SC voxels. Thus, when compared to the conventional analysis, the PNM revealed biologically plausible and expected areas of activity in the left SC, and reduced the number of non-SC voxels marked as active. Activity in the right SC was well localised for both the conventional and PNM analysis, with only 6 and 13 voxels within the non-SC midbrain respectively. No other clusters were revealed outside of the SC, which is in line with our hypothesis.

## Discussion

By applying a novel registration pathway optimised for the midbrain, and an established method to reduce physiological noise, we have shown it is possible to improve the capability of high-resolution fMRI to record task-induced activity within the human midbrain in a small group of subjects (N = 10). We tested these optimised methods on a simple visual paradigm and showed that they improve the accuracy with which activity is localised to midbrain structures, and also reveal biologically plausible activity that in a simpler analysis was obscured due to the presence of un-modelled physiological noise.

### Registration

The four-step registration pathway showed a significant improvement over both the conventional two-step registration used previously in midbrain studies (Krebs et al., 2010; Sigalovsky and Melcher, 2006; Zhang et al., 2006), and the three-step registration pathway recommended by and typically used within the FSL analysis pipeline. The optimised pathway improved the co-localisation of the RN across participants on the standard brain template. There was a greater overlap of the RN between participants and a significantly greater overlap of the group RN mask with the location of the RN on the standard brain template. There were also fewer voxels falsely identified as belonging to the RN when the optimised four-step registration pathway was used. The reasons for the improvement of this method were twofold. First, intermediate scans maintained the contrast of nuclei within the midbrain until the data had been transferred onto a high-resolution structural scan. Thus, the registration algorithms could utilise both the midbrain edges and the borders of the internal midbrain nuclei and maintain registration accuracy throughout this region. Second, the use of weighting volumes prioritised midbrain registration and ignored areas of the brain that suffered from EPI distortion or were of no interest. Although only one nucleus within the midbrain was used in the assessment of the midbrain registration accuracy, it is reasonable to assume that increased accuracy would persist throughout the entire midbrain, as no special efforts were made during the registration optimisation to co-localise the RN over and above any other midbrain area. The use of nonlinear algorithms (FNIRT) ensured accurate registration throughout the whole-brain for the transformation of high-resolution T1w structural images to the standard T1 template, so this step did not require optimisation for the midbrain.

Optimising the registration in this way improved the accuracy with which the midbrain nuclei of individual participants' co-localised on the standard brain template. This reduced any blurring of a genuine signal that would occur with poor co-localisation, and afforded greater confidence when assigning activity to a specific structure.

The optimised registration pathway overcame many of the challenges of midbrain registration, and permitted group level analyses across participants on a standard template, rather than relying on ROI analyses. The use of this type of group level analysis permits voxel-wise comparisons that may reveal regions of activity that are not within pre-determined ROIs, and are not predicted by a prior hypothesis. It also provides the opportunity to detect patterns of activity within an area that have previously been regarded as a single ROI, as functional units of the midbrain may not match the anatomical subdivisions used to define ROIs.

### Physiological noise

Modelling and removing noise with the PNM significantly improved the ability to measure a BOLD signal from the human midbrain. Including cardiac, respiratory, interaction and heart rate regressors in the GLM removed structured physiological noise from the data and led to an increased number of voxels that were demonstrated to be significantly active in response to the visual stimuli. Activity in the left SC, which had been masked by physiological noise, was revealed by the PNM analysis. The number of false positive voxels was also reduced in the PNM analysis, compared to the conventional analysis. This result is consistent with electrophysiological recordings from the SC in awake primates, which show that each colliculus holds a representation of the contralateral visual field (Goldberg and Wurtz, 1972). The visual stimuli used in this study covered both sides of the visual field, and so would have resulted in activity in both the left and right SC.

Unlike RETROICOR, the PNM has been specifically optimised for the spinal cord and brainstem (Harvey et al., 2008). Due to the noise characteristics in these regions, higher harmonics of the physiological cycles explain significant noise in the data, so are included in the PNM. This is the first time the PNM has been tested with such high-resolution scans. It is essential to use such small voxels in midbrain fMRI, as it allows accurate localisation of activity to a specific nucleus, and reduces partial volume effects. However, as voxel size decreases, so does the SNR of the fMRI data, making it more difficult to detect real signal (Edelstein et al., 1986). This study has shown that it is possible to measure a midbrain signal at high resolution, and that using the PNM increases the effective temporal SNR (Cohen-Adad et al., 2010; Hutton et al.), and permits detection of significant effects in a relatively small group size of ten subjects. We demonstrate that the PNM is effective within the SC, and the PNM has previously been shown to be effective within the spine (Brooks et al., 2008; Cohen-Adad et al., 2010) and the motor areas of the brainstem (Harvey et al., 2008). In addition it has been shown that physiological noise in the brainstem is widespread and spatially non-specific (Harvey et al., 2008). Thus it is likely that the PNM will be effective in other areas of the midbrain, although this will require further investigation.

Recently, an alternative method has been developed to remove physiological noise from fMRI data using reference voxels that are assumed to contain signal unrelated to stimulation to model noise in the time series data (de Zwart et al., 2008). This method has been applied to SC data (Wall et al., 2009), using an area of the cerebellum as a reference region. However this method carries the risk of removing “functional” signal from the data, or conversely not removing all the physiological noise. If the noise properties vary between the reference and task regions, noise removal will not be optimal. Whilst it may be safe to assume the physiological noise is similar between the posterior midbrain and the adjacent anterior cerebellum, this assumption would be less valid in more anterior portions of the midbrain. Thus a single reference region cannot adequately model noise throughout the whole midbrain. The PNM, however, models noise on a voxel by voxel basis, and therefore accounts for local variations.

### Limitations

The benefits of optimising fMRI for the midbrain, using the methods described here, also brings with it practical costs. In terms of data acquisition, the added T2w scan requires an additional six minutes of scan time. It is also worth noting that it is not a trivial task to setup the ECG equipment for each participant, and the use of pulse oximetry would provide a less invasive and faster pre-scan setup.

In addition to these practical issues, there are also disadvantages in limiting the FOV. Whilst it is necessary to do this to allow higher resolution scanning at a reasonable temporal resolution, this does limit the region that can be investigated. This is further restricted by the optimised registration, which focuses on the midbrain, potentially at the expense of regions outside this area. This means that subcortical and cortical regions that the midbrain is interacting with at a network level cannot be investigated. The PNM has previously been tested in the brain and spinal cord (Cohen-Adad et al., 2010) and shown to effectively increase the temporal SNR in both regions. Thus it may be possible to investigate cortical and midbrain networks using the PNM. However this would not be optimised for the midbrain to the same extent as the methods presented here, as the larger voxels required for whole-brain coverage would be less reliable at assigning activity to a specific midbrain nucleus, due to their small size and tightly packed arrangement. It would also be difficult to achieve optimum registration at both the level of the cortex and the midbrain simultaneously. Thus the role of such focused and high-resolution midbrain fMRI presented here will be to identify regions of activity that are induced in certain tasks with greater reliability, which can then be combined with whole-brain studies to look for network interactions.

Finally, the sample size used here was small, and although this was sufficient for these purposes, a larger sample size may be required for future studies, particularly if more complex tasks are administered that might evoke more subtle neuronal responses.

### Summary

The methodology presented here improves on previous techniques used to measure BOLD responses in the superior colliculi. Earlier studies did not attempt to optimise midbrain registration, and either used an ROI approach to extract signal (DuBois and Cohen, 2000; Schneider and Kastner, 2005; Schneider and Kastner, 2009; Sylvester et al., 2007; Wall et al., 2009), or relied on standard registration techniques (Krebs et al., 2010). Many studies have not attempted to reduce the effect of physiological noise (DuBois and Cohen, 2000; Krebs et al., 2010; Schneider and Kastner, 2005; Schneider and Kastner, 2009), whilst others have only corrected for cardiac effects (Sylvester et al., 2007) or applied corrections that are specific to only one area of the midbrain (Wall et al., 2009).

The methods outlined here can be used to further investigate properties of the SC, such as the functional difference between the superficial and deep layers and retinotopic organisation (Cynader and Berman, 1972). Further research will investigate if these techniques are effective throughout the midbrain. This could include, for instance, the imaging of the pars compacta of the SN and the adjacent ventral tegmental area in relation to reward and salience. Previous attempts to image these dopaminergic nuclei have either used cardiac gating (D'Ardenne et al., 2008), RETROICOR (Guitart-Masip et al., 2011), or conventional fMRI (Aron et al., 2004; Chase and Clark, 2010; Murray et al., 2008; Waltz et al., 2009; Wittmann et al., 2005).

In conclusion, we report a methodology that optimises midbrain fMRI, allowing accurate localisation of group derived activity and improved ability to detect a signal from the surrounding noise. This methodology is automated and replicable, and uses standard analysis tools.

The following are the supplementary materials related to this article.

Supplementary Table 1 The MRI acquisition parameters for the functional EPI, whole-brain EPI and structural T2 scans.

## References

[bb0005] Aron A.R., Shohamy D., Clark J., Myers C., Gluck M.A., Poldrack R.A. (2004). Human midbrain sensitivity to cognitive feedback and uncertainty during classification learning. J. Neurophysiol..

[bb0010] Basbaum A.I., Fields H.L. (1984). Endogenous pain control systems: brainstem spinal pathways and endorphin circuitry. Annu. Rev. Neurosci..

[bb0015] Beckmann C.F., Jenkinson M., Smith S.M. (2003). General multilevel linear modeling for group analysis in FMRI. Neuroimage.

[bb0020] Brainard D.H. (1997). The psychophysics toolbox. Spat. Vis..

[bb0025] Brooks J.C.W., Beckmann C.F., Miller K.L., Wise R.G., Porro C.A., Tracey I., Jenkinson M. (2008). Physiological noise modelling for spinal functional magnetic resonance imaging studies. Neuroimage.

[bb0030] Chang C., Cunningham J.P., Glover G.H. (2009). Influence of heart rate on the BOLD signal: the cardiac response function. Neuroimage.

[bb0035] Chase H.W., Clark L. (2010). Gambling severity predicts midbrain response to near-miss outcomes. J. Neurosci..

[bb0040] Cohen-Adad J., Gauthier C.J., Brooks J.C.W., Slessarev M., Han J., Fisher J.A., Rossignol S., Hoge R.D. (2010). BOLD signal responses to controlled hypercapnia in human spinal cord. Neuroimage.

[bb0045] Cynader M., Berman N. (1972). Receptive-field organization of monkey superior colliculus. J. Neurophysiol..

[bb0050] D'Ardenne K., McClure S.M., Nystrom L.E., Cohen J.D. (2008). BOLD responses reflecting dopaminergic signals in the human ventral tegmental area. Science.

[bb0055] Dagli M.S., Ingeholm J.E., Haxby J.V. (1999). Localization of cardiac-induced signal change in fMRI. Neuroimage.

[bb0060] de Zwart J.A., Gelderen P., Fukunaga M., Duyn J.H. (2008). Reducing correlated noise in fMRI data. Magn. Reson. Med..

[bb0065] Drayer B., Burger P., Darwin R., Riederer S., Herfkens R., Johnson G.A. (1986). MRI of brain iron. AJR Am. J. Roentgenol..

[bb0070] DuBois R.M., Cohen M.S. (2000). Spatiotopic organization in human superior colliculus observed with fMRI. Neuroimage.

[bb0075] Dunckley P., Wise R.G., Fairhurst M., Hobden P., Aziz Q., Chang L., Tracey I. (2005). A comparison of visceral and somatic pain processing in the human brainstem using functional magnetic resonance imaging. J. Neurosci..

[bb0080] Edelstein W.A., Glover G.H., Hardy C.J., Redington R.W. (1986). The intrinsic signal-to-noise ratio in NMR imaging. Magnetic resonance in medicine. Off. J. Soc. of Magn. Reson. Med..

[bb0085] Friese S., Hamhaber U., Erb M., Kueker W., Klose U. (2004). The influence of pulse and respiration on spinal cerebrospinal fluid pulsation. Invest. Radiol..

[bb0090] Glover G.H., Li T.Q., Ress D. (2000). Image-based method for retrospective correction of physiological motion effects in fMRI: RETROICOR. Magnetic resonance in medicine. Off. J. Soc. Magn. Reson. Med..

[bb0095] Goldberg M.E., Wurtz R.H. (1972). Activity of superior colliculus in behaving monkey. I. Visual receptive fields of single neurons. J. Neurophysiol..

[bb0100] Greitz D., Wirestam R., Franck A., Nordell B., Thomsen C., Stahlberg F. (1992). Pulsatile brain movement and associated hydrodynamics studied by magnetic resonance phase imaging. The Monro-Kellie doctrine revisited. Neuroradiology.

[bb0105] Guimaraes A.R., Melcher J.R., Talavage T.M., Baker J.R., Ledden P., Rosen B.R., Kiang N.Y., Fullerton B.C., Weisskoff R.M. (1998). Imaging subcortical auditory activity in humans. Hum. Brain Mapp..

[bb0110] Guitart-Masip M., Fuentemilla L., Bach D.R., Huys Q.J., Dayan P., Dolan R.J., Duzel E. (2011). Action dominates valence in anticipatory representations in the human striatum and dopaminergic midbrain. J. Neurosci..

[bb0115] Harvey A.K., Pattinson K.T.S., Brooks J.C.W., Mayhew S.D., Jenkinson M., Wise R.G. (2008). Brainstem functional magnetic resonance imaging: disentangling signal from physiological noise. J. Magn. Reson. Imaging.

[bb0120] Hawley M.L., Melcher J.R., Fullerton B.C. (2005). Effects of sound bandwidth on fMRI activation in human auditory brainstem nuclei. Hear. Res..

[bb0125] Hu X., Le T.H., Parrish T., Erhard P. (1995). Retrospective estimation and correction of physiological fluctuation in functional MRI. Magnetic resonance in medicine. Off. J. Soc. Magn. Reson. Med..

[bb0130] Hutton, C., Josephs, O., Stadler, J., Featherstone, E., Reid, A., Speck, O., Bernarding, J., Weiskopf, N., The impact of physiological noise correction on fMRI at 7T. Neuroimage 57, 101–112. 10.1016/j.neuroimage.2011.04.018 PMC3115139 21515386

[bb0135] Jenkinson M., Bannister P., Brady M., Smith S. (2002). Improved optimization for the robust and accurate linear registration and motion correction of brain images. Neuroimage.

[bb0140] Jenkinson M., Smith S. (2001). A global optimisation method for robust affine registration of brain images. Med. Image Anal..

[bb0145] Klein A., Andersson J., Ardekani B.A., Ashburner J., Avants B., Chiang M.-C., Christensen G.E., Collins D.L., Gee J., Hellier P., Song J.H., Jenkinson M., Lepage C., Rueckert D., Thompson P., Vercauteren T., Woods R.P., Mann J.J., Parsey R.V. (2009). Evaluation of 14 nonlinear deformation algorithms applied to human brain MRI registration. Neuroimage.

[bb0150] Klose U., Strik C., Kiefer C., Grodd W. (2000). Detection of a relation between respiration and CSF pulsation with an echoplanar technique. J. Magn. Reson. Imaging.

[bb0155] Krebs R.M., Woldorff M.G., Tempelmann C., Bodammer N., Noesselt T., Boehler C.N., Scheich H., Hopf J.M., Duzel E., Heinze H.J., Schoenfeld M.A. (2010). High-field FMRI reveals brain activation patterns underlying saccade execution in the human superior colliculus. PLoS One.

[bb0160] Liston A.D., Lund T.E., Salek-Haddadi A., Hamandi K., Friston K.J., Lemieux L. (2006). Modelling cardiac signal as a confound in EEG-fMRI and its application in focal epilepsy studies. Neuroimage.

[bb0165] Murray G.K., Corlett P.R., Clark L., Pessiglione M., Blackwell A.D., Honey G., Jones P.B., Bullmore E.T., Robbins T.W., Fletcher P.C. (2008). Substantia nigra/ventral tegmental reward prediction error disruption in psychosis. Mol. Psychiatry.

[bb0170] Naidich T.P., Duvernoy H.M., Delman B.N., Sorensen A.G., Kollias S.S., Haacke E.M. (2009). Duvernoy's Atlas of the Human Brain Stem and Cerebellum.

[bb0175] Napadow V., Dhond R., Kennedy D., Hui K.K.S., Makris N. (2006). Automated brainstem co-registration (ABC) for MRI. Neuroimage.

[bb0180] Napadow V., Dhond R., Park K., Kim J., Makris N., Kwong K.K., Harris R.E., Purdon P.L., Kettner N., Hui K.K. (2009). Time-variant fMRI activity in the brainstem and higher structures in response to acupuncture. Neuroimage.

[bb0185] Nichols T.E., Holmes A.P. (2002). Nonparametric permutation tests for functional neuroimaging: a primer with examples. Hum. Brain Mapp..

[bb0190] Pattinson K.T., Mitsis G.D., Harvey A.K., Jbabdi S., Dirckx S., Mayhew S.D., Rogers R., Tracey I., Wise R.G. (2009). Determination of the human brainstem respiratory control network and its cortical connections in vivo using functional and structural imaging. Neuroimage.

[bb0195] Pelli D.G. (1997). The VideoToolbox software for visual psychophysics: transforming numbers into movies. Spat. Vis..

[bb0200] Piché M., Cohen-Adad J., Nejad M.K., Perlbarg V., Xie G., Beaudoin G., Benali H., Rainville P. (2009). Characterization of cardiac-related noise in fMRI of the cervical spinal cord. Magn. Reson. Imaging.

[bb0205] Poncelet B.P., Wedeen V.J., Weisskoff R.M., Cohen M.S. (1992). Brain parenchyma motion: measurement with cine echo-planar MR imaging. Radiology.

[bb0210] Raj D., Anderson A.W., Gore J.C. (2001). Respiratory effects in human functional magnetic resonance imaging due to bulk susceptibility changes. Phys. Med. Biol..

[bb0215] Schiller P.H., Koerner F. (1971). Discharge characteristics of single units in superior colliculus of the alert rhesus monkey. J. Neurophysiol..

[bb0220] Schneider K.A., Kastner S. (2005). Visual responses of the human superior colliculus: a high-resolution functional magnetic resonance imaging study. J. Neurophysiol..

[bb0225] Schneider K.A., Kastner S. (2009). Effects of sustained spatial attention in the human lateral geniculate nucleus and superior colliculus. J. Neurosci..

[bb0230] Schultz W., Dayan P., Montague P.R. (1997). A neural substrate of prediction and reward. Science.

[bb0235] Sigalovsky I.S., Melcher J.R. (2006). Effects of sound level on fMRI activation in human brainstem, thalamic and cortical centers. Hear. Res..

[bb0240] Smith S.M. (2002). Fast robust automated brain extraction. Hum. Brain Mapp..

[bb0245] Sylvester R., Josephs O., Driver J., Rees G. (2007). Visual FMRI responses in human superior colliculus show a temporal-nasal asymmetry that is absent in lateral geniculate and visual cortex. J. Neurophysiol..

[bb0250] Topolovec J.C., Gati J.S., Menon R.S., Shoemaker J.K., Cechetto D.F. (2004). Human cardiovascular and gustatory brainstem sites observed by functional magnetic resonance imaging. J. Comp. Neurol..

[bb0255] Tracey I., Ploghaus A., Gati J.S., Clare S., Smith S., Menon R.S., Matthews P.M. (2002). Imaging attentional modulation of pain in the periaqueductal gray in humans. J. Neurosci..

[bb0260] Wall M.B., Walker R., Smith A.T. (2009). Functional imaging of the human superior colliculus: an optimised approach. Neuroimage.

[bb0265] Waltz J.A., Schweitzer J.B., Gold J.M., Kurup P.K., Ross T.J., Salmeron B.J., Rose E.J., McClure S.M., Stein E.A. (2009). Patients with schizophrenia have a reduced neural response to both unpredictable and predictable primary reinforcers. Neuropsychopharmacology.

[bb0270] Wittmann B.C., Schott B.H., Guderian S., Frey J.U., Heinze H.-J., Düzel E. (2005). Reward-related FMRI activation of dopaminergic midbrain is associated with enhanced hippocampus-dependent long-term memory formation. Neuron.

[bb0275] Woolrich M.W., Behrens T.E., Beckmann C.F., Jenkinson M., Smith S.M. (2004). Multilevel linear modelling for FMRI group analysis using Bayesian inference. Neuroimage.

[bb0280] Worsley K.J., Evans A.C., Marrett S., Neelin P. (1992). A three-dimensional statistical analysis for CBF activation studies in human brain. J. Cereb. Blood Flow Metab..

[bb0285] Zhang W.-T., Mainero C., Kumar A., Wiggins C.J., Benner T., Purdon P.L., Bolar D.S., Kwong K.K., Sorensen A.G. (2006). Strategies for improving the detection of fMRI activation in trigeminal pathways with cardiac gating. Neuroimage.

