# Supplementary material for: Identification and characterisation of midbrain nuclei using optimised functional magnetic resonance imaging
Source: Neuroimage. 2012 Jan 16;59(2-5):1230–8. doi: 10.1016/j.neuroimage.2011.08.016 (PMC3236997; doi:10.1016/j.neuroimage.2011.08.016)
Supplement: Supplementary Table 1 — The MRI acquisition parameters for the functional EPI, whole-brain EPI and structural T2 scans. [file mmc1.doc]

Supplementary Table 1

|  | Functional EPI | Wholebrain EPI | T2 |
| --- | --- | --- | --- |
| Field of View (mm) | 200(FH) x 200(RL) x 25(AP) | 200(FH) x 200(RL) x 220(AP) | 240 (AP) x 180 (RL) x 180 (FH) |
| In- slice resolution (mm) | 1.5 x 1.5 | 1.5 x 1.5 | 1.15x1.15 |
| Slice thickness (mm) | 1.5 | 1.5 | 1.2 |
| Reconstruction Matrix | 144 | 144 | 256 |
| Sense Factor | 2 (RL) | 2 (RL) | 2 (RL) |
| Number of slices | 16 | 147 | 150 |
| Slice gap (mm) | 0 | 0 | 0 |
| Slice Orientation | Coronal | Coronal | Transverse |
| Fold-over direction | RL | RL | RL |
| Fat shift direction | L | L | P |
| Slice scan order | Interleaved | Interleaved | Interleaved |
| Scan Mode | MS | MS | MS |
| Technique | FFE | FFE | SE |
| Flip angle | 90 | 90 | 90 |
| Repetition Time (ms) | 1600 | 14333 | 3000 |
| Echo Time (ms) | 44 | 44 | 80 |
| Shim | PB volume shim | PB volume shim | Default |
| NSA | 1 | 3 | 1 |
| Fast Imaging Mode | - | - | TSE |
| Shot mode | - | - | Multishot |
| TSE Factor | - | - | 15 |
| Rest Slab | - | - | 1 |
| type | - | - | Parallel |
| Thickness (mm) | - | - | 60 |
| Position | - | - | Feet |
| Gap | - | - | Default |
| Power | - | - | 1 |
